# A long-term survey of *Serratia* spp. bloodstream infections revealed an increase of antimicrobial resistance involving adult population

**DOI:** 10.1128/spectrum.02762-23

**Published:** 2024-01-17

**Authors:** Blanca Pérez-Viso, Marta Hernández-García, Concepción M. Rodríguez, Miguel D. Fernández-de-Bobadilla, María Isabel Serrano-Tomás, Ana María Sánchez-Díaz, José Avendaño-Ortiz, Teresa M. Coque, Patricia Ruiz-Garbajosa, Rosa del Campo, Rafael Cantón

**Affiliations:** 1 Servicio de Microbiología, Hospital Universitario Ramón y Cajal and Instituto Ramón y Cajal de Investigación Sanitaria (IRYCIS), Madrid, Spain; 2 CIBERINFEC. Instituto de Salud Carlos III, Madrid, Spain; JMI Laboratories, North Liberty, Iowa, USA

**Keywords:** bacteremia, bloodstream infections, *Serratia *spp., retrospective study, whole genome sequencing

## Abstract

*Serratia* spp. is a well-recognized pathogen in neonates; however, limited data are available in adults. We studied microbiological and clinical characteristics of *Serratia* spp. causing bloodstream infections (BSI) in our institution (January 2005–July 2020). Overall, 141 BSI episodes affecting 139 patients were identified and medical records reviewed. Antimicrobial susceptibility was recovered from our informatics system and 118 isolates from 116 patients were available for further microbiological studies. Whole genome sequencing (WGS) was completed in 107 isolates. Incidence of *Serratia* BSI was 0.3/1000 overall admissions (range 0.12–0.60), with maximum prevalence (27 episodes, 19.1%) during 2017–2018. Relevant patients’ clinical characteristics were 71.9% ≥60 years (*n* = 100), with high comorbidity rates (49%, ≥2), 23 (74.2%) of them died within 1 month of the BSI episode. WGS identified all isolates as *Serratia marcescen*s when Kraken bioinformatics taxonomic tool was used despite some which were identified as *Serratia nematodiphila* (32/118) or *Serratia ureilytica* (5/118) by matrix-assisted laser desorption/ionization time-of-flight mass spectrometry. Nevertheless, when using MASH distance, *Serratia nevei* (63/107), *S. ureilytica* (38/107), and *S. marcescens* (6/107) were assigned. Carbapenemase (*bla*_VIM-1_) and extended-spectrum β-lactases (ESBL) (*bla*_SHV-12_) genes were found in seven and three isolates, respectively, one of them expressing both genes. The worldwide-disseminated IncL/M scaffold plasmid was identified in six VIM producers. Four genotypes were established based on their virulence factors and resistome. *Serratia* spp. emerged as a relevant nosocomial pathogen causing BSI in elderly patients in our hospital, particularly in recent years with a remarkable increase in antibiotic resistance. ESBL and carbapenemases production related to plasmid dissemination are particularly noteworthy.

**IMPORTANCE:**

*Serratia* spp. is the third most frequent pathogen involved in outbreaks at neonatal facilities and is primarily associated with bacteremia episodes. In this study, we characterized all causing bloodstream infection (BSI) in patients admitted to our hospital during a 16-year period (2005–2020). Despite having no neonatal intensive care unit in our hospital, this study revealed that *Serratia* spp. is a relevant pathogen causing BSI in elderly patients with high comorbidity rates. A significant increase of antimicrobial resistance was detected over time, particularly in 2020 and coinciding with the coronavirus disease (COVID-19) pandemic and nosocomial spread of multidrug-resistant *Serratia* spp. isolates. extended-spectrum β-lactases and carbapenemases genes associated with plasmid dissemination, typically detected in other Enterobacterales species, were also identified, reinforcing the role of *Serratia* spp. in the antimicrobial resistance landscape. Additionally, this work highlights the need to reclassify the species of *Serratia*, since discrepancies were observed in the identification when using different tools.

## INTRODUCTION

Bloodstream infections (BSIs), mainly caused by Gram-negative pathogens, are a major cause of morbidity and mortality worldwide, being also a priority for the World Health Organization (https://www.paho.org/es/70-asamblea-mundial-salud), with variations in incidence and epidemiology between countries (1–3). Globally, *Serratia* is low represented among the BSI pathogens, although it has a significant burden in the neonatal population, especially among preterm infants (4, 5). However, the microbiological and clinical features of *Serratia* spp. and infections caused by this pathogen have been little explored in the adult population, with a lack of a trend analysis of its antibiotic resistance. *Serratia* species harbor intrinsic antibiotic-resistant genes, such as those responsible for the production of the chromosomal AmpC β-lactamases, which when hyperproduced confers resistance to extended-spectrum cephalosporins (6). It also carries other intrinsic resistant determinant affecting aminoglycosides and polypeptides (7, 8). The acquisition of plasmid-mediated resistance mechanisms, such as those involving extended-spectrum β-lactases (ESBLs) and carbapenemases, has also been reported (9).

In the present work, we aimed to assess the incidence of *Serratia* spp. causing BSI in our institution, which do not have a neonatal intensive care unit (ICU), over a 16-year period (2005–2020), characterizing its distinctive epidemiological, clinical, and microbiological features.

## RESULTS

### Epidemiology of *Serratia* spp. causing bloodstream infections at Ramón y Cajal University Hospital

**Fig 1:**
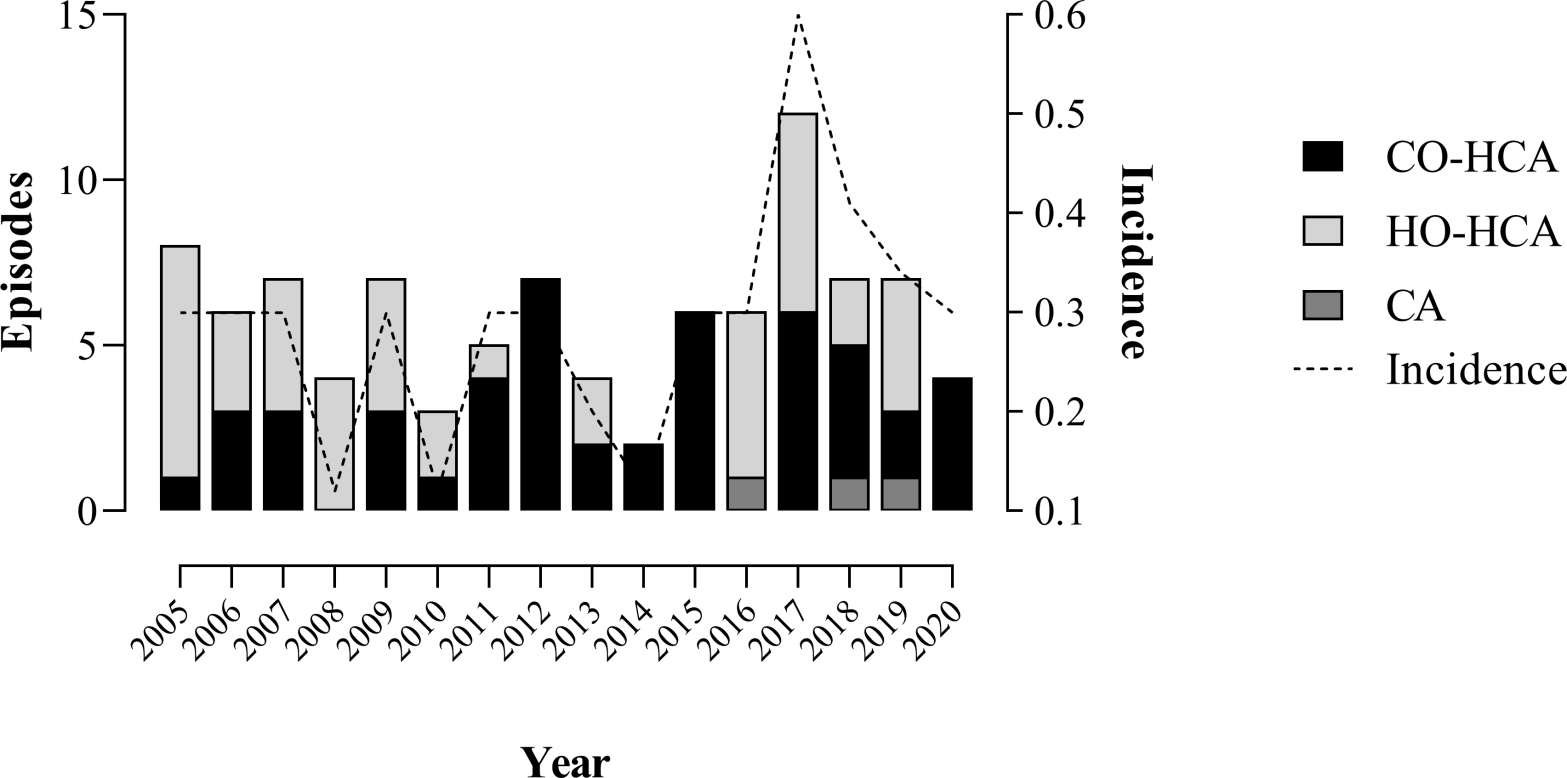
Incidence of *Serratia* BSI (episodes/1,000 overall hospitalizations) during the period studied and occurrence of hospital-onset healthcare-associated (HO-HCA), community-onset healthcare care-associated (CO-HCA), and community-acquired (CA) episodes.

Overall, 141 BSI episodes caused by *Serratia* spp. were identified between 2005 and 2020, two patients had two different isolates in separate periods (>30 days) and were considered as independent. The total incidence of BSIs caused by *Serratia* spp. in our hospital was 0.30/1,000 overall admissions (range 0.12–0.60) (Fig. 1), with the highest prevalence in 2017 and 2018 (14/141 and 13/141 episodes, respectively, that corresponds to 19.1% of the entire period). *Serratia* spp. was co-isolated with other microorganisms in 19 episodes (13.5%), mostly with *Enterococcus faecalis* (*n* = 4) and *Enterobacter cloacae* (*n* = 3). Catheters (40/141, 28.4%), surgical wounds (16/141, 11.3%), and the urinary (14/141, 9.9%) and respiratory tract (13/141, 9.2%) were the most frequent sources of the infection, although in 41 cases (29.1%), the origin could not be determined. Matrix-assisted laser desorption/ionization time-of-flight mass spectrometry (MALDI-TOF MS) identified *Serratia marcescens* as the most represented species, with 81/118 (68.6%) of the total BSIs; however, *Serratia nematodiphila* (32/118, 27.1%) and *Serratia ureilytica* (5/118, 4.2%) were also identified.

#### Patients’ epidemiological characteristics

BSI were mostly associated with men (89/139, 64.0%). The patients’ ages ranged from 21 days to 97 years (average 68.3 years ± 17.3). Age distribution was clearly shifted toward older patients; 100/139 (71.9%) patients were aged ≥60 years, 33/139 (23.7%) between 20 and 59 years, and only 6 (4.3%) patients between 0 and 19 years.

At sampling, 35/139 (25.1%) patients were in the emergency department, and 47/139 (33.9%) were hospitalized in various medical wards [oncology (*n* = 12), internal medicine (*n* = 6), cardiology (*n* = 7), nephrology (*n* = 7), and others (*n* = 15)], 26 (18.7%) were in surgical areas, and finally, 31 (22.3%) in ICUs. Based on the epidemiological data of all *Serratia* BSI episodes (*n* = 141), 87 (61.7%) were classified as hospital-onset healthcare-associated (HO-HCA), (51 (36.2%) as community-onset healthcare care-associated (CO-HCA), and 3 (2.1%) as community acquired (CA) (Fig. 1).

Patients exhibited high rates of comorbidities, 80 of them (57.6%) with two or more comorbidities highlighting hypertension and oncologic diseases. It should be noted that 31 patients died (22.3%) within the first month of BSI diagnosis, 23 (74.2%) of them had ≥60 years. (Table 1). After bacteremia diagnosis, most of the patients were treated in monotherapy with fluoroquinolones or carbapenems alone. Combination therapy was used in 25.5% of the patients. No significant differences were obtained regarding the antimicrobial treatment and clinical outcome (Table S1). We did not detect any case of selection of AmpC hyperproduction associated with the treatment with cephalosporins.

**TABLE 1:** Demographic and clinical characteristics of patient’s population with bloodstream infections due to *Serratia* spp.^*a*^

	Patients (*n* = 139)	Survivors (*n* = 108)	Non-survivors (*n* = 31)	*P*-value
Age – years	69.5 ± 17.34			
>60 years – *n* (%)	100 (71.9)	77 (71.3)	23 (74.2)	0.3631
Sex, male – *n* (%)	89 (64.02)	68 (63)	21 (67.7)	0.6764
Comorbidities – *n* (%)				
Diabetes	16 (11.5)	14 (13)	2 (6.5)	0.3622
Dyslipidemia	18 (13)	16 (14.8)	2 (6.5)	0.2460
Hypertension	44 (31.7)	33 (30.6)	11 (35.4)	0.8386
CVD^*b*^	13 (9.3)	8 (7.4)	5 (16.2)	0.3057
Digestive	14 (10.1)	13 (12)	1 (3.2)	0.1908
Immunodeficiency	9 (6.5)	7 (6.4)	2 (6.5)	>0.9999
COPD	11 (8)	6 (5.5)	5 (16.1)	0.1348
Oncologic disease	38 (27.3)	27 (25)	11 (35.4)	0.3953
Two or more	80 (57.6)	63 (58.3)	17 (54.8)	0.6840

^*a*^ Data expressed as mean ± SD or *n* (percentage). *P*-values in Fisher’s exact test between survivors and non-survivors are shown.

^*b*^ CVD, cardiovascular disease; COPD, chronic obstructive pulmonary disease.

#### Antimicrobial susceptibility and resistance phenotypes

The antimicrobial susceptibility profile is shown in Table 2. The last 6 years of the study (2015–2020) showed the highest rates of antimicrobial resistance (Table S2). Overall, tobramycin was the aminoglycoside that presented the highest rate of resistance from this group (48.3%). Cefotaxime had a resistance rate of 15.6%, and the resistant rate for cefepime was 7.8%. Carbapenems (imipenem, meropenem, and ertapenem) were the antimicrobial group with the greatest rate of susceptibility (96.5%, 96.5%, and 94.3%, respectively).

**TABLE 2:** Antimicrobial susceptibility profile of the 141 *S*. *marcescens* isolates causing BSI^*a*^

	TZP^*b*^^,*c*^	CTX	FEP	ATM	IPM	ETP	MEM	GEN	AMK	TOB	CIP
R	12 (8.5)	22 (15.6)	11 (7.8)	12 (8.5)	5 (3.5)	9 (6.4)	5 (4.2)	9 (6.4)	7 (5)	67 (47.5)	21 (14.9)
I	3 (2.1)	2 (1.4)	1 (0.7)	0 (0)	0 (0)	0 (0)	0 (0)	0 (0)	0 (0)	0 (0)	0 (0)
S	126 (88.4)	117 (83)	129 (91.5)	129 (91.5)	136 (96.5)	132 (93.6)	113 (95.8)	132 (93.6)	134 (95)	74 (52.5)	121 (85.1)

^*a*^ Data expressed as *n* (percentage). Meropenem results are regarding 118 isolates.

^*b*^ TZP, piperacillin/tazobactam; CTX, cefotaxime; FEP, cefepime; ATM, aztreonam; IPM, imipenem; ETP, ertapenem; MEM, meropenem; GEN, gentamicin; AMK, amikacin; TOB, tobramycin; CIP, ciprofloxacin.

^*c*^ S, susceptible; I, susceptible, increased exposure; R, resistant. Data expressed as *n* (percentage).

ESBL production was corroborated in two isolates, carbapenemase production in six isolates, and both enzymes in one isolate. A positive result in the phenotypic AmpC induction test was detected in 87 out of 97 cefotaxime susceptible isolates (89.7%) (Fig. S1). The induction positivity was not observed in ESBL or carbapenemase producers, even in the former with cefepime, due to the expanded cephalosporin resistance conferred by these enzymes. Moreover, according to the AmpC induction test and not considering the result of ESBL and carbapenemase producers, 12 isolates of all 118 isolates (10.2%) hyperproduced the AmpC enzymes, all of them being susceptible to cefepime.

#### Bacterial typing, whole genome sequencing (WGS), and bioinformatics analysis

The WGS process was successfully completed in 107 of the 118 isolates. Eleven isolates were excluded for the bioinformatics analysis due to low sequence quality.

##### Genome characteristics and bacterial classification

The median genome size of our isolates was 5 Mb–5.1 Mb, with a G + C content of 59.5% and an average of 4,727 protein-coding sequences. Genomic information based on contig size ≥500 bp is detailed in Table S3. Interestingly, Kraken identified all the isolates as *S. marcescens*, despite some of these isolates previously identified as *S. nematodiphila* (32/118) or *S. ureilytica* (5/118) by MALDI-TOF. Nevertheless, using PATO tool, which is based on MASH distance*, Serratia nevei* (58.9%, 63/107) was the most prevalent species detected, followed by *S. ureilytica* (35.5%, 38/107) and *S. marcescens* (5.6%, 6/107). Interestingly, only five isolates were identified as *S. marcescens* with both WGS tools and MALDI-TOF MS and only two *S*. *ureilytica* were identified with both PATO and Kraken. Results comparing the three identification tools are detailed in Table S4.

##### Population structure

**Fig 2:**
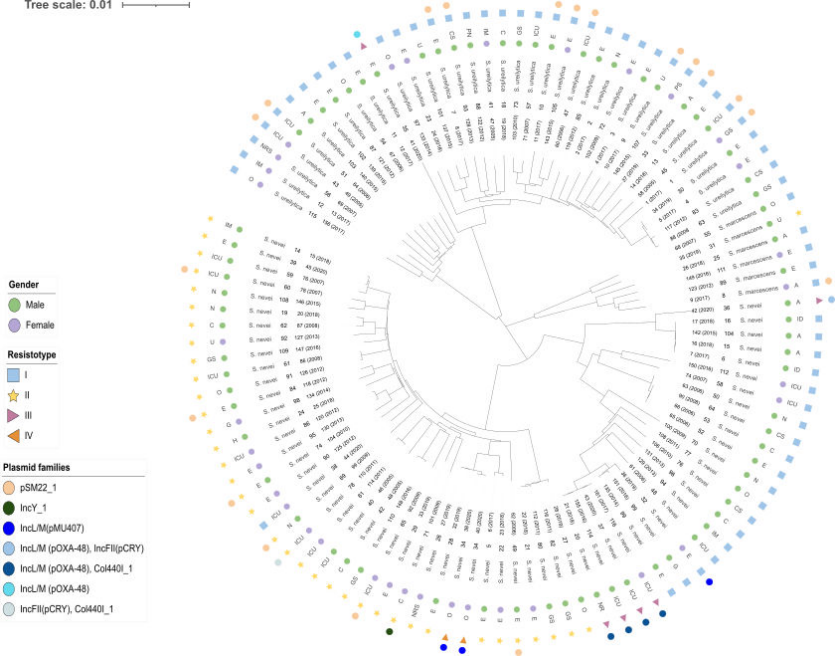
Similarity tree construction by iTOL with the whole genome of the 107 BSI *Serratia* isolates. O, oncology; IM, internal medicine; NRS, neurosurgery; ICU, intensive care unit; A, anesthesia; E, emergency; U, urology; CS, cardiac surgery; PN, pneumology; C, cardiology; GS, general surgery; ID, infectious diseases; N, nephrology; PS, plastic surgery; NR, neurology, G, gastroenterology; H, hematology. Number of isolate with year of isolation are shown in brackets.

A similarity tree was constructed with the 107 isolates (Fig. 2) revealing that our *Serratia* spp. BSI isolates did not present a clonal origin. Moreover, we did not find any clear proximity in the isolates according to their year of isolation. Nevertheless, they were mainly grouped in resistotypes I and II. Distribution of resistotypes was irrespective of *Serratia* spp. identification.

##### Antibiotic resistance genes and resistome classification

Isolates carried *bla*_SRT-1_ (*n* = 72/107; 67.3%) or *bla*_SST-1_ (*n* = 35/107; 32.7%) genes related to inducible chromosomal AmpC β-lactamase in *Serratia*. The bioinformatic analysis confirmed the presence of other genes conferring resistance to aminoglycosides (*aac*6-lc, *aad*A1, *aph*A15, *str*A, and *str*B), tetracyclines (*tet*41), chloramphenicol (*cat*A1, *cat*B2, and *cml*B1), trimethoprim/sulfamethoxazole (*dfr*B1), macrolides (*mph*E and *msr*E), sulfonamides (*sul*1), fluoroquinolones (*qnr-*S1), and β-lactamases (*bla*LAP-2, *bla*_SHV-12_ and *bla*_VIM-1_). Plasmidic AmpC genes were not detected.

The carbapenemase-producing isolates (*n* = 7) carried the *bla*_VIM-1_ gene, and except for one isolate (isolate 101), all *bla*_VIM-1_ carriers expressed the same co-resistance pattern: *sul*1, *cat*A1, *cat*B2, *aad*A1, *aac*6-lc, *dfr*B1, *mph*E, *msr*E, *bla*_SRT-1_, and *bla*_VIM-1_. All but one (isolate 41, *S. ureilytica*) were identified as *S. nevei*.

The *bla*_SHV-12_ ESBL gene was identified in three isolates, one of them also harboring *bla*_VIM-1_. Two of these isolates also exhibited fluoroquinolone and aminoglycoside resistance. Interestingly, except for one isolate (isolate 101), all ESBL and carbapenemase producers were detected in the last 4 years (2017–2020).

Based on these results, four resistotypes were established regarding resistance traits involving both intrinsic and acquired resistance and genes: I (*aac*6-Ic, *bla*_SST-1_, *tet*41) and II (*aac*6-Ic, *bla*_SRT-1_) were the most frequently represented (62/107, 58% and 37/107, 34,6%, respectively). In addition to aminoglycoside resistance and β-lactamase encoding genes representing resistotype II (*n* = 35), two strains, one harboring chloramphenicol resistance gene (*cml*B1) and other one (isolate 101) in which co-production of SHV-12 and VIM-1 was identified (*bla*_SHV-12_, *bla*_VIM-1_), were added to this resistotype as they are closely related in the similarity tree. Resistotype III included six isolates in which carbapenemase production was also detected (*aac*6-Ic, *aad*A1, *bla*_SRT-1_, *bla*_VIM-1_, *tet*41, *sul*1, *cat*A1, *cat*B2, *dfr*B1, *mph*E, *msr*E), and resistotype IV is represented by two strains in which ESBL *bla*_SHV-12_ production was identified (*aac*6-Ic, *bla*_SRT-1_, *bla*_SHV-12_, *bla*_LAP-2_, and *qnr*-S1).

##### Virulome

The virulence genes detected in our isolates were related to flagella proteins (*fli*P, *fli*M, *fli*G, *fli*A, *flg*H), protein regulators of chemotaxis (*che*Y) and heat-stable enterotoxin (*ast*A); *fli*G and *fli*M (107/107, 100%) were the most frequently represented, followed by *che*Y (64/107, 60%).

##### Plasmid gene content

Plasmid replicon content was identified in 28% (30/107) of the isolates. The families detected belonged to various incompatibility group (Inc) replicons: IncL/M, IncFII, IncY, and IncF [IncL/M (pMU407), IncFII (pCRY), IncY_1, pSM22_1, respectively]. In addition, the worldwide spread of IncL/M (pOXA-48) was detected in six isolates, with a coverage and identity of 100%, in all cases related to those strains in which *bla*_VIM-1_ production was detected (genotype VI). Regarding isolate 101, the only one co-producing ESBL and carbapenemase, the IncY_1 plasmid was identified.

Col440I_1, belonging to the Col-plasmid family, was detected in five strains, four of them in VIM-1 producers associated to IncL/M (pOXA-48).

## DISCUSSION

Bacteremia is associated with high mortality, ranging between 10% and 30% depending on patient risk factors, the origin of the infection, and antimicrobial treatment. Nosocomial infections due to *Serratia* spp. have increased, highlighting its pathogenic role, particularly in ICUs. *Serratia* spp. constitutes the third most frequent pathogen involved in outbreaks at neonatal facilities and is primarily associated with bacteremia episodes (10, 11). In our series, 22.3% overall mortality within 1 month of BSI diagnosis was observed, although they were vulnerable given their elderly age and clinical conditions.

Despite having no neonatal ICU in our hospital, *S. marcescens* represents a frequent pathogen causing BSI in our patients (0.30/1,000 hospitalizations, range 0.12–0.60), 71.9% of them with ≥60 years and 22.3% admitted in ICU. In our context, as well as reported by other authors, the most frequent origins of *Serratia* bacteremia are the genitourinary tract, surgical wounds, respiratory tract, and intravascular catheters; however, in up to 29.1% (41/141) of the cases, the source is unknown (10). This figure is similar to previous studies, in which the source of the infection was not determined in a high proportion of the bacteremia episodes.

Although Kraken, a taxonomic sequence classification system, confirmed that all isolates were identified as *S. marcescens*, MASH distance tool also revealed a majority presence of *S. nevei* and *S. ureilytica* besides a minority presence of *S. marcescens*. Also, MALDI-TOF MS identified some of them as *S. nematodiphila* and *S. ureilytica*. Differences on identification when using different tools have been described in this study. Nevertheless, MALDI-TOF is currently the reference method in clinical microbiology laboratories. These species are closely related to *S. marcescens*, and phylogenetic studies have proposed re-classifying them as *S. marcescens* based on their core genome phylogeny (8, 12). Identification obtained with “classifier” function of PATO tool was employed to search for the closet reference species in the NCBI (National Center for Biotechnology Information), highlighting that the recently described *S. nevei* is also closely related to *S. marcescens,* demonstrating the complexity in *Serratia* spp. taxonomy.

The increase in antimicrobial resistance is one of the most important public health challenges in our society, and its spread is mainly driven by horizontal transfer of resistance genes (13). Intrinsically, *S. marcescens* has an inducible chromosomal β-lactamase, which confers resistance to aminopenicillins, first- and second-generation cephalosporins, remaining susceptible to third- and fourth-generation cephalosporins, monobactams, and carbapenems (14). *S. marcescens* is characterized by its rapid acquisition of antibiotic resistance, mainly due to mutations in regulatory genes affecting derepression of its inducible chromosomal AmpC β-lactamase but also due to plasmid acquisition. In our series, we did not document cases of selection of AmpC hyperproduction after treatment with cephalosporins. This situation coincided with the low risk for significant AmpC production indicated by Tamma et al. (15).

Isolates from our study exhibited high resistance rates, particularly during the last 6 years (2015–2020). This is also the studied period with the highest number of *Serratia* spp. isolates (*n* = 67), with resistance rate to cefotaxime (12.8%) and cefepime (7.8%), reaching up to 71.4% in the nine isolates from 2020. This period was also coincident with the description of carbapenemase producing *S. marcescens* in our hospital (16). Tobramycin is the aminoglycoside that showed the highest resistance rate in this period due to the chromosomal *aac*6-Ic gene that is related to this intrinsic resistance profile, although other aminoglycosides as gentamicin were not affected (17).

A major limitation of our study could be that the antimicrobial susceptibility results were retrospectively retrieved from the laboratory records and were obtained with the automated systems routinely used in the clinical microbiology laboratory of our hospital. Nevertheless, these data represent real-life susceptibility testing results reported to the clinicians. Broth microdilution, the reference standard method, was only performed prospectively for meropenem, while other newly developed molecules were not tested. Notably, the latest isolates recovered in 2020 exhibited the highest antibiotic resistance rates, demonstrating a significant increase in antimicrobial resistance over time, which also coincides with the first year of the coronavirus disease (COVID-19) pandemic and overuse of antimicrobials in the hospital setting (18).

On the other hand, the spread of antimicrobial resistance mechanisms such as carbapenemases is typically associated with Enterobacterales species, mostly *Escherichia coli* and *Klebsiella pneumoniae*. However, recent studies performed at our institution confirmed the contribution of other species, such as *S. marcescens* and *Enterobacter* spp (16, 19). In our study, WGS showed that all isolates harbored intrinsic or acquired genes conferring resistance to various antimicrobials (defining six distinct genotypes), including β-lactamases. The *bla*_SST-1_ and *bla*_SRT-1_ genes encoding for the inducible AmpC β-lactamases in *S. marcescens* were found in all isolates. Their phenotypic induction was observed in 89.7% of cefotaxime-susceptible tested isolates (87 out of 97 isolates). This was not the case in ESBL or carbapenemase producers in which these enzymes hid the potential positive result in the induction test. Even using cefepime, it cannot be ruled out as these resistance mechanisms might also make difficult the correct interpretation of the AmpC induction phenotypic test (15, 20, 21). ESBL and carbapenemase producers (except the isolate that produced both enzymes) were mostly recovered in the last 4 years of the study. Moreover, we did not detect the *bla*_SME_ gene, a class A carbapenemase gene previously found inserted in the *S. marcescens* chromosome (22).

The virulome characterization showed a narrower trait. Only virulence factors related to flagella, proteins of chemotaxis, and enterotoxin were present. Although *Serratia* species are opportunistic pathogens, their mechanisms of invasion and pathogenesis remain to be elucidated (7, 23).

Various plasmid incompatibility groups were detected, and replicons belonging to the IncL/M family were the most frequent. The WGS comparative analysis confirmed that six *Serratia* spp. BSI isolates (VIM-1 producers recovered during the last years of studied period) carried the IncL/M plasmid, harboring the same class I integron with *bla*_VIM-1_ carbapenemase gene, which had been found in previous studies from our institution (13, 16), suggesting a considerable ability to persist (24).

In conclusion, this retrospective study helps to understand the role of *S. marcescens* as a pathogen in the antimicrobial resistance landscape of BSIs, particularly in recent years, with the acquisition of ESBL and carbapenemase genes associated with plasmid dissemination. Moreover, we provide new insights of BSIs due to this pathogen in the elderly population.

## MATERIALS AND METHODS

### Study design, bacterial isolates selection, and patients’ characteristics

Between January 2005 and July 2020, a total of 363,168 blood cultures (median 22,698 per year) were processed at Ramón y Cajal University Hospital, a tertiary-level public health center with 1,155 beds that serves approximately 600,000 inhabitants in the northern area of Madrid (Spain). A total of 141 of *Serratia* spp. BSIs episodes affecting 139 patients were detected in this period. To avoid the study of duplicate isolates, only one positive sample per patient and BSI episode was considered. Nevertheless, isolates obtained 1 month apart from the same patient, which occurs in two patients, were also separately considered. Finally, 118 of the isolates, recovered from 116 patients, were available from the strain collection of the Microbiology Department at the HRyC for further studies. All available isolates (*n* = 118), originally identified by biochemical tests at the time of their isolation, were re-identified by MALDI-TOF MS, library IVD 4194, and RUO 4274 (Bruker-Daltonics, Germany), and the latter with WGS and bioinformatics analysis (see below). Clinical and epidemiological data were retrospectively reviewed from the clinical chart of all patients (*n* = 141). For comorbidity, we considered medical conditions that normally influence clinical outcomes such as diabetes or hypertension that exists simultaneously but independently of those associated with BSI.

Bacteremia episodes were classified as HO-HCA, CO-HCA, or CA, according to Friedman’s criteria (25). Briefly, episodes that occurred at least ≥48 h after hospitalization with no symptoms of infection at hospital admission were considered to be HO-HCA. Episodes within 48 h of admission were considered as CO-HCA or CA if the patient had had recent contact with a healthcare setting or not, respectively. The study was approved by the hospital’s Ethics Committee (reference 136/22).

### Antimicrobial susceptibility

Antimicrobial susceptibility MIC values (12 antimicrobials) obtained by broth microdilution in automated systems (Wider, Soria Melguizo, 2005–2011 or MicroScan, Beckman Coulter, 2011–2020) were retrieved from our laboratory informatics system from all cases (*n* = 141). In addition, meropenem was latter tested with standard manual broth microdilution in all available isolates (26). Due to several modification of breakpoints over the study period, the EUCAST 2020 criteria (https://www.eucast.org/) were used for the interpretation of MIC data presented in the manuscript.

### Phenotypic and molecular antimicrobial resistance gene detection

The screening to detect ESBL production was performed with the double disc diffusion synergy test (27). Inducible AmpC production was tested with disc approximation test, using cefoxitin and imipenem as inducers, with cefotaxime, ceftazidime and cefepime (28). Carbapenemase production was phenotypically determined with the KPC/MBL/OXA-48 Confirm Kit (Rosco Diagnostica, Denmark).

### Whole genome sequencing and bioinformatics analysis

Total DNA extraction was performed from 2 mL of bacterial exponential growth cultures using the Chemagic DNA Bacterial External Lysis Kit (PerkinElmer, USA), and WGS was performed with the Illumina-Novaseq 6000 platform (OGC, Oxford, UK) with 2 × 150 bp paired-end reads. Quality control and sequence filtering employing FastQC v.0.11.9 (https://www.bioinformatics.babraham.ac.uk/projects/fastqc/) tools were performed. Short reads were assembled with SPAdes v3.13.1 (29), and genome assembly evaluation was performed by QUAST v5.1 (30). Bacterial identification was confirmed with the Taxonomic Sequence Classification System Kraken v.1.1.1 (31). and PATO package in R, calculating the MASH distance (maximum distance = 0.06) with all reference and representative genomes from NCBI Refseq database (32). The draft genomes were annotated by Prokka v.1.14.6 (33), and antimicrobial resistance, virulence genes, and plasmids were identified and characterized with Abricate v.1.0.1, employing the ResFinder, ARG-ANNOY, VFDB, and Plasmidfinder databases (threshold: 92% identity; 90% coverage) (34). The Mash v.2.3 and iTOL applications were used to generate and trace a similarity tree based on a neighbor-joining algorithm (35). The complete genome sequences were deposited at DDBJ/ENA/GenBank under the project number PRJNA898137 (JAQSOV000000000-JAQSSX000000000).

### Statistical analysis

Differences of clinical characteristics, comorbidities, and antibiotic therapy treatment between survivor and non-survivors were checked by Fisher’s exact test. *P*-values of <0.05 were considered significant at a 95% CI. Statistical analyses were performed with IBM SPSS 23 (Armonk, NY, USA) and GraphPad Prism 8 (San Diego, CA, USA) software. The sample size (*n*) as well as the specific statistical test in each analysis are shown in the tables.

## SUPPLEMENTAL MATERIAL

Figure S1 spectrum.02762-23-s0001.pdf Characteristics of Serratia BSI isolates regarding AmpC production.

Table S1 spectrum.02762-23-s0002.docx Antibiotic treatment administered to patients considering total number of episodes (n = 141).

Table S2 spectrum.02762-23-s0003.docx Antimicrobial resistance rates in Serratia BSI isolates over years. Data expressed as percentage of resistant strains in each period.

Table S3 spectrum.02762-23-s0004.xlsx Genomics characteristics of *S. marcescens* bacteremia isolates.

Table S4 spectrum.02762-23-s0005.xlsx Bacterial identification results depending on tool used.

ASM does not own the copyrights to Supplemental Material that may be linked to, or accessed through, an article. The authors have granted ASM a non-exclusive, world-wide license to publish the Supplemental Material files. Please contact the corresponding author directly for reuse.
