# Correction for Pérez-Viso et al., “A long-term survey of *Serratia* spp. bloodstream infections revealed an increase of antimicrobial resistance involving adult population”

**DOI:** 10.1128/spectrum.02425-24

**Published:** 2024-11-11

**Authors:** Blanca Pérez-Viso, Marta Hernández-García, Concepción M. Rodríguez, Miguel D. Fernández-de-Bobadilla, María Isabel Serrano-Tomás, Ana María Sánchez-Díaz, José Avendaño-Ortiz, Teresa M. Coque, Patricia Ruiz-Garbajosa, Rosa del Campo, Rafael Cantón

## AUTHOR CORRECTION

Volume 12, no. 2, e02762-23, 2024, https://doi.org/10.1128/spectrum.02762-23. We have detected that Table S2 in the supplemental material contains errors (percentages and *P* values). These errors do not affect the article itself, but some of the mistaken percentages of Table S2 were referred to inaccurately.

Page 4, Results: “Overall, tobramycin was the aminoglycoside…” should read “… from this group (47.5%).”

“Carbapenems (imipenem, meropenem, and ertapenem) were…” should read “… greatest rate of susceptibility (96.5%, 95.8%, and 93.6%, respectively).”

Page 7, Discussion: “This is also the studied period with the highest number of *Serratia* spp. isolates…” should read “… cefotaxime (26.9%), and cefepime (16.4%) …”

Supplemental material: Table S2 should appear as shown in the revised supplemental file in this author correction.

## SUPPLEMENTAL MATERIAL

Table S2 spectrum.02425-24-s0001.docx Antimicrobial resistance rates in Serratia BSI isolates over years. Data expressed as percentage of resistant strains in each period. Meropenem results are regarding 118 isolates.

ASM does not own the copyrights to Supplemental Material that may be linked to, or accessed through, an article. The authors have granted ASM a non-exclusive, world-wide license to publish the Supplemental Material files. Please contact the corresponding author directly for reuse.

