# Supplementary figures and images for: A long-term survey of Serratia spp. bloodstream infections revealed an increase of antimicrobial resistance involving adult population
Source: Microbiol Spectr. 2024 Jan 17;12(2):e02762-23. doi: 10.1128/spectrum.02762-23 (PMC10846012; doi:10.1128/spectrum.02762-23)

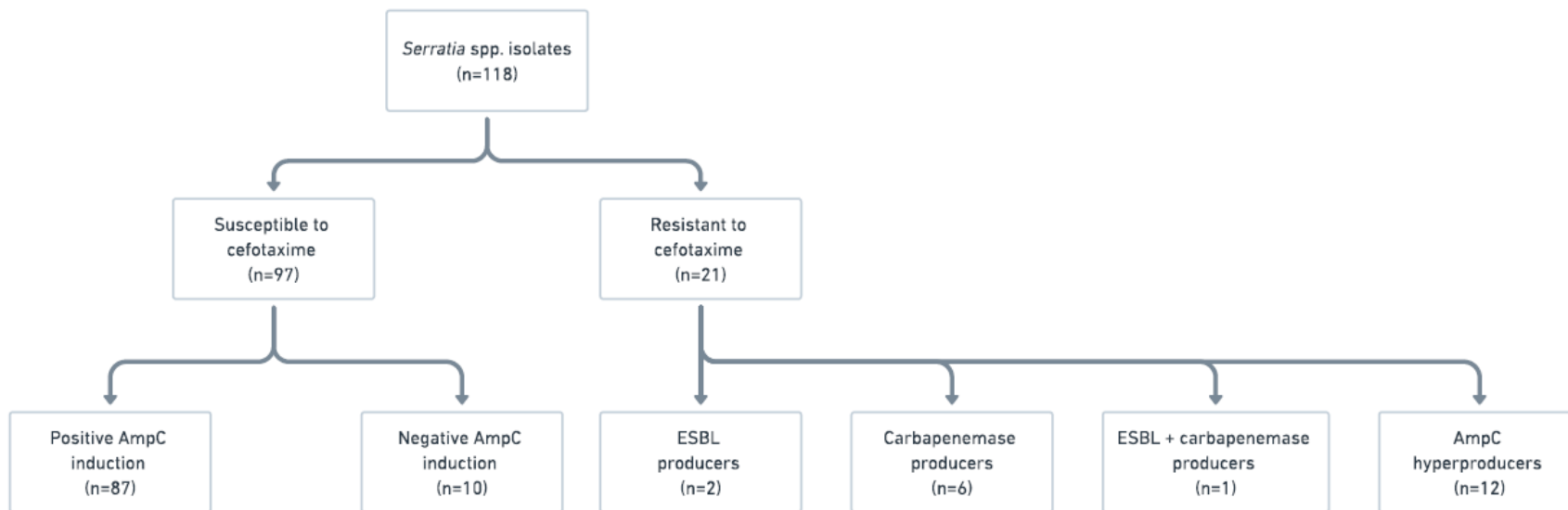

**Figure S1.** Characteristics of *Serratia* BSI isolates regarding AmpC production.

Supplement: Figure S1 — Characteristics of Serratia BSI isolates regarding AmpC production. [file spectrum.02762-23-s0001.pdf]
