# Supplementary material for: A long-term survey of Serratia spp. bloodstream infections revealed an increase of antimicrobial resistance involving adult population
Source: Microbiol Spectr. 2024 Jan 17;12(2):e02762-23. doi: 10.1128/spectrum.02762-23 (PMC10846012; doi:10.1128/spectrum.02762-23)
Supplement: Table S1 — Antibiotic treatment administered to patients considering total number of episodes (n = 141). [file spectrum.02762-23-s0002.docx]

**Table S1**. Antibiotic treatment administered to patients considering total number of episodes (n=141)

| **Antimicrobial treatment** | Episodes (n=141) | Survivors  (n=110) | Non-survivors  (n=31) | p-value |
| --- | --- | --- | --- | --- |
| No antimicrobial therapy | 17 (12.1) | 9 (8.2) | 8 (25.8) | 0.0131 |
| Monotherapy | 88 (62.4) | 74 (67.3) | 14 (45.2) | 0.0351 |
| Penicillins | 16 (18.2) | 13 (17.6) | 3 (21.4) | 0.7134 |
| Cephalosporins | 9 (10.2) | 9 (12.2) | 0 | 0.3477 |
| Fluoroquinolones | 22 (25) | 20 (27) | 2 (14.3) | 0.3384 |
| Carbapenems | 19 (21.6) | 16 (21.6) | 3 (21.4) | >0.9999 |
| Other classes | 22 (25) | 16 (21.6) | 6 (42.9) | 0.1046 |
| Combination therapy | 36 (25.5) | 27 (24.5) | 9 (29) | 0.6441 |
