# Supplementary material for: A long-term survey of Serratia spp. bloodstream infections revealed an increase of antimicrobial resistance involving adult population
Source: Microbiol Spectr. 2024 Jan 17;12(2):e02762-23. doi: 10.1128/spectrum.02762-23 (PMC10846012; doi:10.1128/spectrum.02762-23)
Supplement: Table S2 — Antimicrobial resistance rates in Serratia BSI isolates over years. Data expressed as percentage of resistant strains in each period. [file spectrum.02762-23-s0003.docx]

**Table S2**. Antimicrobial resistance rates in *Serratia* BSI isolates over years Data expressed as percentage of resistant strains in each period.

| **Antimicrobial agent** | **2005-2009**  **(n=42)** | **2010-2014**  **(n=32)** | **2015-2020**  **(n=67)** | **p-value** |
| --- | --- | --- | --- | --- |
| **TZP** | 0.0% | 0.7% | 7.8% | 0.2729 |
| **CTX** | 0.7% | 2.1% | 12.8% | 0.1713 |
| **FEP** | 0.0% | 0.0% | 7.8% | 0.1346 |
| **ATM** | 0.7% | 1.4% | 6.4% | 0.2225 |
| **IPM** | 0.0% | 0.0% | 3.5% | 0.0006 |
| **ETP** | 0.7% | 0.0% | 5.7% | 0.0301 |
| **MEM** | 0.0% | 0.0% | 3.5% | 0.0006 |
| **GEN** | 0.7% | 0.7% | 5.0% | 0.0301 |
| **AMK** | 0.0% | 0.0% | 5.0% | 0.0062 |
| **TOB** | 12.1% | 8.5% | 27.7% | <0.0001 |
| **CIP** | 3.5% | 2.8% | 8.5% | 0.2781 |

TZP, piperacillin/tazobactam; CTX, cefotaxime; ; FEP, cefepime; ATM, aztreonam; IPM, imipenem; ETP, ertapenem; MEM, meropenem; GEN, gentamicin; AMK, amikacin; TOB, tobramycin; CIP, ciprofloxacin.
