# Supplementary material for: Correction for Pérez-Viso et al., “A long-term survey of Serratia spp. bloodstream infections revealed an increase of antimicrobial resistance involving adult population”
Source: Microbiol Spectr. 2024 Nov 11;12(12):e02425-24. doi: 10.1128/spectrum.02425-24 (PMC11619376; doi:10.1128/spectrum.02425-24)
Supplement: Table S2 — Antimicrobial resistance rates in Serratia BSI isolates over years. Data expressed as percentage of resistant strains in each period. Meropenem results are regarding 118 isolates. [file spectrum.02425-24-s0001.docx]

**Table S2**. Antimicrobial resistance rates in *Serratia* BSI isolates over years. Data expressed as percentage of resistant strains in each period. Meropenem results are regarding 118 isolates.

| **Antimicrobial agent** | **2005-2009**  **(n=42)*** | **2010-2014**  **(n=32)*** | **2015-2020**  **(n=67)*** | **p-value** |
| --- | --- | --- | --- | --- |
| **TZP** | 0.0% | 3.1% | 16.4% | 0.0053 |
| **CTX** | 2.4% | 9.4% | 26.9% | 0.0015 |
| **FEP** | 0.0% | 0.0% | 16.4% | 0.0014 |
| **ATM** | 2.4% | 3.1% | 14.9% | 0.0341 |
| **IPM** | 0.0% | 0.0% | 7.5% | 0.0571 |
| **ETP** | 2.4% | 0.0% | 11.9% | 0.0339 |
| **MEM** | 0.0% | 0.0% | 8.8% | 0.0612 |
| **GEN** | 2.4% | 3.1% | 10.4% | 0.1698 |
| **AMK** | 0.0% | 0.0% | 10.4% | 0.0171 |
| **TOB** | 38.1% | 40.6% | 56.7% | 0.1120 |
| **CIP** | 11.9% | 12.5% | 17.9% | 0.6307 |

*Regarding meropenem: 2005-2009 (n=33), 2010-2014 (n=28) and 2015-2020 (n=57). TZP, piperacillin/tazobactam; CTX, cefotaxime; FEP, cefepime; ATM, aztreonam; IPM, imipenem; ETP, ertapenem; MEM, meropenem; GEN, gentamicin; AMK, amikacin; TOB, tobramycin; CIP, ciprofloxacin. p-value is shown in Chi-square test.
